# Multi-Modal Fusion of Routine Care Electronic Health Records (EHR): A Scoping Review

**DOI:** 10.3390/info16010054

**Published:** 2025-01-15

**Authors:** Zina Ben-Miled, Jacob A. Shebesh, Jing Su, Paul R. Dexter, Randall W. Grout, Malaz A. Boustani

**Affiliations:** 1 Phillip M. Drayer Department of Electrical and Computer Engineering, Lamar University, Cherry Building, Beaumont, TX 77705, USA; 2 Department of Electrical and Computer Engineering, School of Engineering and Technology, Indiana University Purdue University at Indianapolis, 723 W. Michigan Street, Indianapolis, IN 46202, USA; 3 Indiana University School of Medicine, Indiana University, 340 W 10th St, Indianapolis, IN 46202, USA; 4 Regenstrief Institute, Inc., 1101 W 10th St, Indianapolis, IN 46202, USA

**Keywords:** multi-modal fusion, electronic health records, machine learning, transformers, modality

## Abstract

**Background:**

Electronic health records (EHR) are now widely available in healthcare institutions to document the medical history of patients as they interact with healthcare services. In particular, routine care EHR data are collected for a large number of patients. These data span multiple heterogeneous elements (i.e., demographics, diagnosis, medications, clinical notes, vital signs, and laboratory results) which contain semantic, concept, and temporal information. Recent advances in generative learning techniques were able to leverage the fusion of multiple routine care EHR data elements to enhance clinical decision support.

**Objective:**

A scoping review of the proposed techniques including fusion architectures, input data elements, and application areas is needed to synthesize variances and identify research gaps that can promote re-use of these techniques for new clinical outcomes.

**Design:**

A comprehensive literature search was conducted using Google Scholar to identify high impact fusion architectures over multi-modal routine care EHR data during the period 2018 to 2023. The guidelines from the PRISMA (Preferred Reporting Items for Systematic Reviews and Meta-Analyses) extension for scoping review were followed. The findings were derived from the selected studies using a thematic and comparative analysis.

**Results:**

The scoping review revealed the lack of standard definition for EHR data elements as they are transformed into input modalities. These definitions ignore one or more key characteristics of the data including source, encoding scheme, and concept level. Moreover, in order to adapt to emergent generative learning techniques, the classification of fusion architectures should distinguish fusion from learning and take into consideration that learning can concurrently happen in all three layers of new fusion architectures (i.e., encoding, representation, and decision). These aspects constitute the first step towards a streamlined approach to the design of multi-modal fusion architectures for routine care EHR data. In addition, current pretrained encoding models are inconsistent in their handling of temporal and semantic information thereby hindering their re-use for different applications and clinical settings.

**Conclusions:**

Current routine care EHR fusion architectures mostly follow a design-by-example methodology. Guidelines are needed for the design of efficient multi-modal models for a broad range of healthcare applications. In addition to promoting re-use, these guidelines need to outline best practices for combining multiple modalities while leveraging transfer learning and co-learning as well as semantic and temporal encoding.

## 1. Introduction

Healthcare is a complex system of patients’ points of care, each offering services within different clinical settings ranging from routine care to specialty clinics and emergency departments. The patient interacts with these services during encounters with healthcare providers which are documented in an Electronic Health Records (EHRs) [1]. Encounter notes document history of present illness, assessments, lab reports, and treatment plans [2]. Other data, such as diagnosis, medications, and procedures, are codified from these notes into a structured format for billing or research purposes [3].

Routine or primary healthcare is the first level of healthcare and should be accessible to all individuals. Therefore, intelligent models that are able to leverage routine care EHR data can reduce the workload of healthcare providers and improve the quality of care for a large number of patients [4,5]. In order to achieve this goal, the models must transform these data into decision support using an adequate learning architecture. Unfortunately, current standard definitions for the elements of routine care EHR data and related model architectures are nebulous.

Routine care EHR data are comprised of heterogeneous data elements which are labeled as modalities in the literature with some inconsistencies. For instance, modality can refer to data sources (e.g., diagnosis, medications, imaging, or clinical notes) [6], data types (e.g., structured or unstructured) [7], or medical concept [8]. In order to address these inconsistencies, modality in the present scoping review is redefined as a triplet (data source, encoding, and concept level).

Multi-modal fusion architectures tend to outperform their uni-modal counterparts [9–11] and are traditionally classified under [12]:

1. Early fusion (data fusion) first combines the modalities into a unified representation which is then used to train a single model.
2. Late fusion (decision fusion) combines the outcomes of submodels trained independently for each modality.
3. Hybrid fusion (intermediate fusion) is the combination of both early and late fusion through gradual intermediate unified representations.

The above classification focuses on the fusion of the encodings of the data from multiple sources making it less applicable to emergent fusion architectures which are increasingly relying on representation learning (RL), pretrained language models (LMs), and deep learning (DL) [13]. As such, under the traditional classification, recent models that use a pretrained LM to encode data or a DL network in the decision layer would systematically fall under the hybrid fusion category.

**Figure 1.**
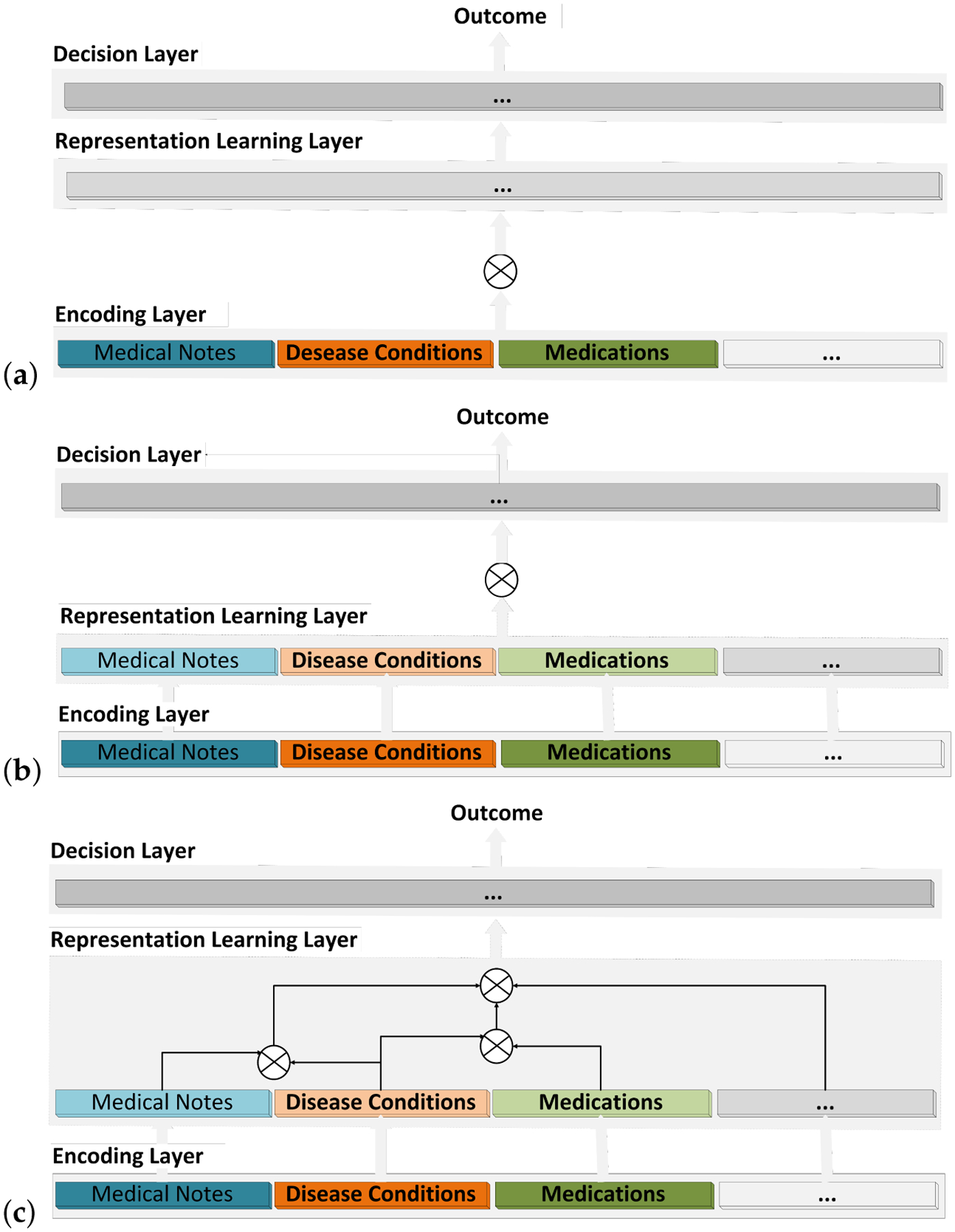
Fusion architectures: (**a**) Encoding; (**b**) Decision; (**c**) Representation Learning.

Emergent fusion architectures should be instead viewed as the stacking of three layers: (a) an encoding layer where raw data are mapped to a numerical space (e.g., clinical notes encoded using a pretrained LM), (b) a representation learning layer where the encoded data are projected onto a latent space, and (c) a decision layer where an outcome is learned from the latent space. Learning can effectively happen in any of these layers. Based on this organization, the classification of fusion architectures is redefined as follows:

1. Encoding fusion (Figure 1a): The encodings of all the input modalities are combined and submitted to a single representation learning model. The latent representation generated by this layer is then used to train the decision layer.
2. Decision fusion (Figure 1b): Independent encoding and representation layers are used for each modality. The latent representations of the modalities are then combined and processed with a single decision layer.
3. Representation learning fusion (Figure 1c): Multiple latent representations are produced using subsets of the modalities. These latent representations are then combined and submitted to the decision layer.

Compared to the traditional classification, the new classification emphasizes the fact that learning can occur in any layer of the fusion architecture to generate encoding, latent representation, or model decision. The three classes are distinguished by the layer in which modalities, in their original or intermediate representation, are combined.

In addition to underscoring the lack of standard definitions for EHR routine care modalities and fusion architecture classification, the present scoping review underlines the need for systematic design strategies for fusion architectures over these modalities. To date, most fusion architectures are handcrafted [14], thereby offering limited guidelines for other clinical outcomes, modalities, and fusion architectures.

The remainder of the manuscript describes the methodology used for the selection of representative articles in support of the present scoping review. This is followed by a detailed description of the characteristics of routine care EHR data and multi-modal fusion architectures as synthesized from the literature review using the revised definitions of modality and fusion classification. The discussion section of the manuscript includes a comparative summary of the strengths and limitations of current fusion architectures and provides directions for future research.

## 2. Methods

The present review was performed in accordance to the PRISMA (Preferred Reporting Items for Systematic Reviews and Meta-Analyses) guidelines. The related protocol is registered under the Open Science Framework (https://osf.io/28y7u/, accessed on 6 January 2025). Google Scholar was selected as the literature database because of its ability to track broader citations and offer wider coverage including conference articles and non-peer reviewed content [15]. This aspect is important given that contributions to the subject of interest are from the fields of engineering and computer science and health and medical sciences. These fields suffer from low coverage in other databases [16].

A search was first conducted to find review manuscripts from 2018 to date. The study period starts in 2018 because this is the publication year of the bidirectional encoder representation from transformers (BERT) [17], which marked a renewed interest in representation learning and fusion models for EHR data [1,18]. The manuscripts collected through this preliminary search confirmed that the objective of the present scoping review was not addressed by previous reviews [1–7,9–14,18–25]; additionally, it allowed the development of an appropriate keyword search lexicon for a subsequent in depth literature search.

### 2.1. Search Queries

Three categories of disjunctive search terms were constructed. The first two are inclusion terms and the third consists of general exclusion terms as follows:

- Clinical: EHR, medical, clinical, biomedical, phenotyping, disease, healthcare, “health record”;
- Technical: multimodal, “multi modal”, transformer, BERT, unstructured, embedding, deep, attention;
- General Exclusion: image, imaging, scan, segmentation, leaf.

Due to the restrictions on the query length imposed by Google Scholar, a subquery was constructed for each clinical term in conjunction with the collection of technical inclusion and general exclusion terms. These subqueries were issued for each year over the period 2018 to 2023. Year to year subqueries were needed to mitigate the 1000 citations limit per query imposed by Google Scholar. The purpose of the general exclusion terms was to exclude studies that primarily focus on imaging (i.e., non-routine care EHR) or are out of scope (e.g., plant disease).

### 2.2. Study Selection

A total number of 13,248 citations, including reviews, were returned by the above queries. Of these, manuscripts that were cited less than 10 times were excluded. This criterion was applied in order to focus on high impact research work that was available for review by peers for about a year resulting in 5814 manuscripts. Additional articles were excluded following a review of the titles and abstracts of the documents. Ambiguous terms (e.g., attention deficit disorder, deep vein thrombosis, and medical attention) contributed the most to the out-of-scope articles.

Core articles (*n* = 102) which either exemplify trends in uni-modal data representations or fusion architectures with a focus on routine care EHR data were identified and organized according to their subjects in Table 1. Others are cited throughout the manuscript.

## 3. Routine Care EHR Data

Routine care EHR captures the patient’s medical history. This section details the differences in EHR data representations and their attributing trade-offs. Particularly, we focus on clinical notes and non-image data. As noted in the literature, a large number of studies already cover imaging data [6]. However, clinical notes are understudied [7].

### 3.1. Demographics

The coding of demographic variables vary from one health care institution to another. For instance, in some EHR systems, race may be limited to values in “White, Black or African American, American Indian or Alaska Native, or Other” while other EHR systems may implement the entire current version of the race dictionary with its 15 categorical values [31]. That said, the distribution of the available training samples for the model may dictate the aggregation of the race variable at a lower cardinality (e.g., “White, Black or African American, Other” or even “White, non-White”) in order to have sufficient samples in each category (TADEL [71], DeepEMC [70]).

Some demographic variable are time-invariant (e.g., race), while other may change over time (e.g., age). Age is calculated from the date of birth of the patient [84], and often converted into a categorical value in 1-year, 5-year, or 10-year intervals, and then one-hot encoded (KG-MTT-BERT [80], MDBERT [46]). Again, the granularity of the quantization depends on the patient’s distribution as well as the target application (e.g., pediatrics [85] versus geriatrics [49]).

The patient’s sex, race, ethnicity, and encounter type (e.g., inpatient or outpatient) have low cardinality and are easily represented using one-hot encoding. Unfortunately, semantic context is lost in this conversion [24]. The text values of sex, race, ethnicity encapsulate semantic meaning related to medical conditions, procedures and treatments (e.g., sex and obstetrics [86]). For this reason, few models encode demographic variables in a text format (e.g., LDAM [72], FarSight [87,88]).

### 3.2. Disease Conditions

In an EHR system, disease conditions are entered in text and then translated into one or more standard taxonomies for different purposes (e.g., insurance claims, research, etc.). In the US, the standard taxonomy for disease conditions is the International Classification of Diseases (ICD) [26]. The translation process (i.e., ICD coding) is manually performed in most healthcare institutions. In fact, one of the important applications of machine learning is to automate ICD coding from clinical text (MSATT-KG [38], DCAN [42], LAAT [43], and MDBERT [46]).

As such, disease conditions can be derived from at least two sources: text or ICD codes [89]. In both cases, the disease modality has high-cardinality, making one-hot encoding impractical. There are two main types of techniques that are used to obtain a lower dimension representation: (1) Combining related diseases into groups using the anatomical or physiological grouping offered by the ICD code [26], the Charlson Comorbidity [29] index, the Elixhauser Comorbidity [30] index, or UMLS [31]; or (2) using a vector representation derived from generative translation via transformer-based LMs such as the ones listed in Table 2.

### 3.3. Medications

Medication also has a high cardinality. As in the case of disease conditions, it can be coded using several taxonomies (e.g., GPI [27], ATC [28], or UMLS) with varying concept levels. For instance, the guiding principle of GPI is the therapeutic use of the medication and the underlying ontology consists of seven hierarchical levels: drug group, drug class, drug subclass, drug base name, drug name, dose form, and GPI name. In contrast, the focus of the ATC taxonomy is on the organ or system of the human body on which the medication acts. It consists of five levels: anatomical or pharmacological group, pharmacological or therapeutic subgroup, chemical, pharmacological, or therapeutic subgroup, and chemical substance.

Surprisingly, despite the inclusion of medications in several multi-modal models, as discussed in the next section, our literature search did not reveal a LM based solely on medications as in the case of disease conditions.

### 3.4. Clinical Notes

Text in clinical notes can be considered as a large collection of variables (e.g., words), where words can have different semantic meanings depending on the context. Encodings for text are limited to a vocabulary of frequent and relevant words called a corpus. Variables derived from text range from characters and word-pieces (tokens) to n-grams [38], or in select cases complete phrases (HAN [44,88]).

Clinical notes have been represented using topic models [87] and in some cases, topic keywords were mapped to a medical ontology such as UMLS [89]. Topic models are application-specific, retain limited semantic or temporal information, and require expert-guided tuning. To avoid these limitations, generative LMs have been introduced [38].

When LMs are used to encode clinical notes, the granularity of the text variables has a direct implication on the generalizability of the encoding. For instance, byte-pair encodings use a limited vocabulary where each entry consists of two characters. This unit size is less likely to produce out-of-vocabulary words even when a LM which is pretrained with a general English corpus is used for a medical application [90]. For larger units, this is not the case. For example, the medical acronyms BMI and A1C are frequent in EHR data but infrequent in a general English corpus.

LMs adapted to the medical domain were shown to deliver improved performance on many downstream tasks (e.g., BioBERT [40]). However, the trade-offs between fine tuning a general LM versus pretraining an LM model from scratch using a medical corpus remain unclear especially when the lack of sufficient in-domain training data is taken into consideration [91,92]. A corpus consisting of a mixture of medical and non-medical data can help address this issue (GatorTron [47]). Table 3 includes a representative list of LMs for clinical notes. Additional examples are described in [93].

### 3.5. Vital Signs and Laboratory Results

Vital signs such as body temperature, blood pressure, and heart rate are collected during in-patient and out-patient encounters [57]. Routine laboratory tests are ordered during annual wellness or in-patient encounters. They include white blood cell counts, cholesterol levels, and culture tests. These data are structured. Depending on the clinical setting, they can be considered as time-in-point measurements or time series [53,67,94]. For example, an elevated temperature in an annual wellness encounter may indicate an infection. In this case, values from previous encounters are not relevant making the variable a time-in-point measurement. By contrast, temperature is recorded frequently and encoded as a time series in an emergency setting and used to monitor the progression of the health status of the patient [68,69]. Even when vital signs and laboratory results are recorded frequently, they can be aggregated into minimum, maximum, and mean values over the observation period to facilitate multi-modal fusion [58]. Our literature search did not reveal a uni-modal LM for this EHR data element.

## 4. Temporal and Semantic Information

As mentioned above, EHR data are collected over multiple clinical encounters. The sequence of these encounters describes the medical history of the patient. To assess the health status of the patient, a healthcare provider performs a chart review. This review entails scanning previous clinical notes, medications, disease conditions, and laboratory tests in the EHR to form a few hypotheses. Deeper investigations further exclude unlikely hypotheses and retain the most likely one. During this process, the temporal and semantic information in the EHR is evaluated and infused with the health provider’s medical knowledge. Once a final hypothesis is constructed, a referral, treatment plan, or additional laboratory tests are ordered. The ultimate machine learning model must be able to replicate this process by capturing both temporal and semantic information [1,23] within and across multiple encounters.

### 4.1. Temporal Information

Clinical encounters document the longitudinal health of a patient including the progression of chronic disease conditions and results of extended treatments (Hi-BEHERT [75]). The frequency of these encounters varies depending on the clinical setting (e.g., outpatient in Patient2vec [55], inpatient in MBERT [76], or emergency in T-GGANN [95]).

**Figure 2.**
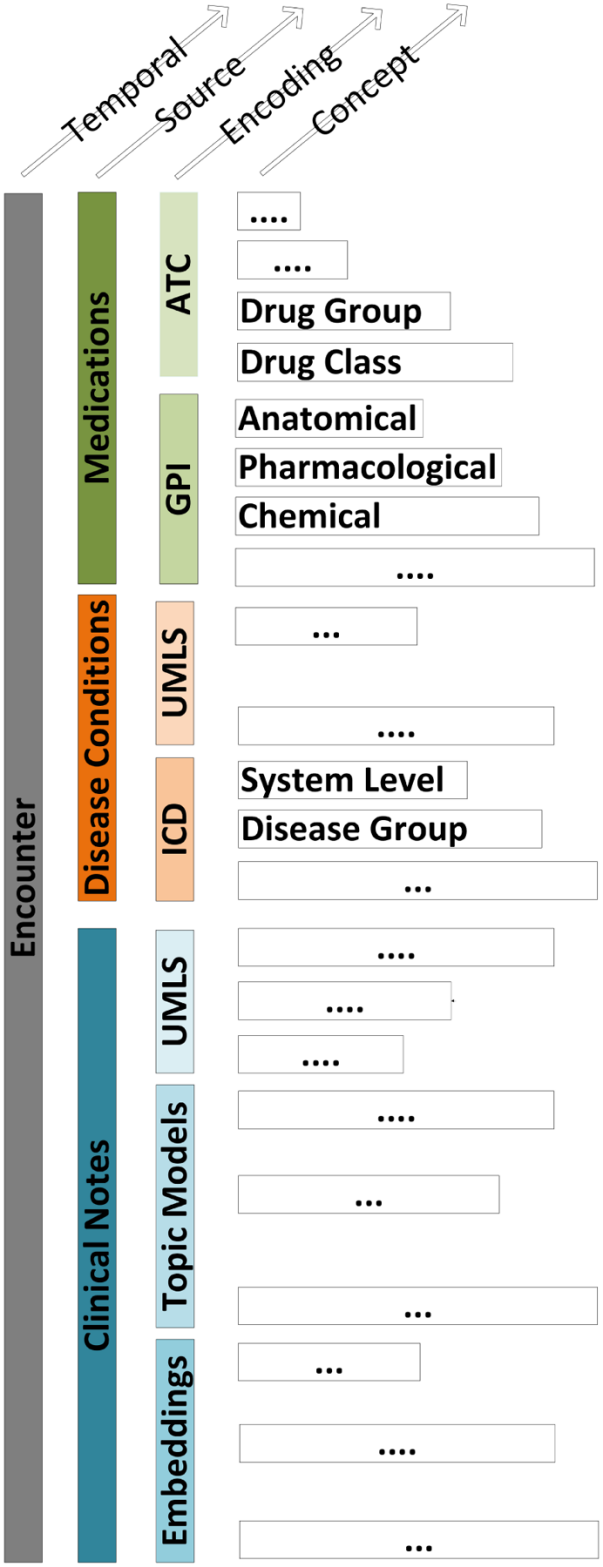
An encounter with representative routine care modalities, encodings, and concept levels.

Temporal information is an integral part of the patient’s EHR since each encounter is timestamped (Figure 2). Several encoding mechanisms avoid temporality by aggregating or binning EHR variables over an observation period suitable for the clinical setting and the target outcome. As such, a sequence of medications or disease conditions is encoded as a list of unordered variables thereby ignoring any precedence relations between these entities [58]. Other encoding attach a token representing the chronological order of the encounters as in BRLTM [65], BiLSTM [59], and CEHR-BERT [66]. This latter technique is similar to the time delay embedding which is used for time series forecasting [96].

Deepr [50] is one of the early LMs that encoded the temporality of EHR variables. The model creates an input embedding consisting of ICD codes, procedures, and the time between patients’ encounters. All input features are concatenated into a single vector. The vector is then encoded into a fixed embedding using a convolutional neural network (CNN). A sliding window over the input is used to capture temporal correlations across EHR data from different encounters.

Med-BERT^1^ [34] also encodes the temporality of disease conditions following the same approach but with a transformer architecture. The model constructs a vector representation of ICD codes and adds an encounter embedding segment which encodes the encounter number. Med-BERT^1^ is pretrained with two tasks: masked language model and the prediction of a patient’s length-of-stay (LOS). Other transformer models followed the same approach when capturing the temporal sequence of encounters (BRLTM [65] and BEHRT [61]). These models concatenate multiple encounters and add an encounter embedding segment that delineates each encounter.

Temporal models have an advantage since they are able to identify inter-encounter correlations [61]. Theoretically, these models can select an individual encounter or multiple encounters to infer clinical decisions as in the case of a chart review. As such, they are able to model the progression of clinical outcomes when exposed to an adequate observation period. Unfortunately, the length of this observation period is hard to determine [7]. For instance, estimating LOS or mortality in an emergency setting only requires a short observation period measured in hours [51,72,84]. In contrast, predicting conversion from mild cognitive impairment to Alzheimer’s disease relies on an extended multi-year observation period [49]. Extended observation periods can exceed the computational limits of current generative modeling techniques [97].

### 4.2. Semantic and Concept Information

In the present review, we adopt the following definitions of semantic and concept. Semantic refers to the meaning of a word within a sequence. Concept refers to a medical concept as defined in an ontology or a taxonomy (Table 1). The relationships among data source, encodings, and concept levels are illustrated in Figure 2 for a representative set of routine care EHR modalities.

The distinction between semantic and concept is important for EHR data. For example, the following two sentences were extracted from the EHRs of two female patients: (1) “Female patient denies meningitis, headaches, confusion”; (2) “She has developed confusion at the hospital”. In both sentences confusion refers to a UMLS concept, whereas semantic information indicates absence and presence of confusion in the first and second sentences, respectively. An encoding that ignores semantic information (e.g., topic models [87,89]) may not be able to ascertain presence or absence of confusion.

The objective of LMs is to capture semantic information. However, the context window is often limited. The self-attention mechanism in the LM architecture has a computational complexity which is quadratic with respect to the input length [98]. This is due to each word-piece (token) having context for every other word-piece in the input. To address this limitation, Longformer introduced a new attention mechanism that scales linearly with input length [99]. This attention mechanism combines a local windowed attention with a dilated sliding window for global context. Large [47] and hierarchical [54] LMs also aim at accommodating extended context windows.

In essence, encoding EHR modalities using topic modeling or medical concepts ignores semantic information. These models are not restricted by long observation periods [87]. In contrast, LMs can encode semantic information but at the expense of an increase in computational complexity for long observation periods. Understanding these trade-offs along with modeling temporal information is an open research problem. These future research directions should consider modality redundancy and the fact that semantic information is more important for some EHR modalities than others.

## 5. Multi-Modal Fusion

As in a chart review, fusion architectures can benefit medical applications because EHR modalities can reinforce one another thereby bolstering correct inferencing [11]. For example, medications are prescribed for specific disease conditions. Clinical notes are summative reports of the health condition of the patient and include disease symptoms or medication efficacy as experienced by the patient (MedM-PLM [77]). Capturing these interactions is the goal of multi-modal fusion (Table 4). This section covers the three fusion architectures as defined in the present scoping review (Figure 1), their advantages, and their limitations when applied to routine care EHR data.

### 5.1. Encoding Fusion

With encoding fusion, features from each modality are combined to form a unified vector, which is processed in the representation learning layer (Figure 1a).

#### 5.1.1. Examples

ATTAIN [57] is an encoding fusion architecture. An LSTM is used to process a series of patient events including vital signs, lab results, procedures, and clinical settings. These data are collected over an observation period of an hour and the target outcome is early prediction of septic shock. The main contribution of ATTAIN is the modeling of temporal information over irregular clinical events. It includes a weighted attention mechanism that takes into consideration the importance of previous events and their relative proximity to the outcome using a time decay function. It would be interesting to validate this architecture for outcomes that require an extended observation period.

The bidirectional representation learning model (BRLTM) [65] is also an encoding fusion transformer architecture trained on multi-modal EHR data. BRLTM combines the following modalities with varying dimensions:

- ICD codes (1131);
- Procedure codes in the Current Procedural Terminology (CPT) format (7048);
- Medications (4181);
- Demographic information (Age, Sex);
- Clinical notes represented as 100 topics generated from a topic model using Latent Dirichlet Allocation (LDA).

BRLTM [65] uses the same temporal embedding scheme as Med-BERT^1^ [34]. A segment is created for each encounter and the concatenation of all the encounters for a patient over the observation period constitutes the input of BRLTM. However, the encounter segment covers not only disease codes but also procedure codes, medication codes, and topics from the clinical notes. Moreover, a separate embedding is created for age, sex, position, and encounter identifier.

BRLTM reported 2% and 10% higher AUCs compared to a representation learning fusion architecture HCET [60] and another encoding fusion architecture BEHRT [61], respectively, for the one-year prediction of three primary diagnoses: myocardial infarction, breast cancer, and liver cirrhosis. Clinical notes were found to have a significant predictive contribution. However, the contributions of age and sex in this architecture were not discussed.

#### 5.1.2. Strengths

The main strengths of encoding fusion, compared to decision fusion, are co-learning [14,22] and temporal encoding [57,61,65]. Additional investigations are needed to confirm these strengths compared to RL fusion [60].

#### 5.1.3. Limitations

Data from some of the modalities are difficult to fuse [23]. Therefore, additional processing and feature engineering are necessary before encoding fusion can be applied. In particular, since the dimensionality of most routine care EHR modalities is high [25], dimension reduction is needed. This can be accomplished by: (1) encoding the modality at a higher concept level following a relevant ontology (Table 1) or (2) encoding the modality using topic modeling, PCA or a pretrained LM.

Moreover, the two methods used to combine the encodings of the modalities are concatenation [57] and summation [61,65]. Summation, or any function that reduces input size (e.g., mean, max, etc.), leads to information loss as the underlying functions are non-invertible.

### 5.2. Decision Fusion

As illustrated in Figure 1b, encoding and representation learning for each modality are performed independently of other modalities in decision fusion. The modality-specific latent representations are combined in the decision layer using a classifier.

#### 5.2.1. Examples

MM-HCR [63] is the decision fusion of two modalities: clinical notes and 17 time series clinical variables. The latent representation of the clinical notes is derived from a semantic module which consists of hierarchical architecture of CNN and RNN layers. The clinical variables are processed with a temporal module that tracks the condition of the patient over time and consists of a two-layers deep bidirectional gated recurrent unit (GRU). In this architecture, semantic information is captured by the first module and temporal information is captured by the second module. The decision fusion of the two modules is accomplished using a dropout layer followed by a sigmoid layer.

MM-HCR was applied to mortality prediction in an emergency setting. The results indicate that the decision fusion model outperformed both the individual clinical notes model and the clinical variables model. However, the performance improvement with respect to the clinical notes model was not significant and the performance of the clinical variables model increased, reaching that of the clinical notes model, when the observation period was extended from 12 to 48 h.

The Holistic AI in Medicine (HAIM [74]) framework is a general decision fusion model that combines multiple data types. HAIM starts by creating latent representations for each data type (i.e., tabular, time series, clinical notes, and images) using appropriate encodings and architectures. For instance, Clinical BERT [39] is used to encode text and a pretrained RNN is used for time series data. All latent representations are then combined with a decision fusion classifier such as SVM, NN, or XGBoost.

When HAIM was applied to multiple applications, the time-series modality was found to be important for temporal tasks such as LOS and 48-h mortality and less important for tasks such as pneumonia and fracture diagnosis. For these latter tasks, the modalities that contributed the most were clinical notes and imaging. The findings of this study were summarized in two main fusion principles: selective contribution and the law of diminishing returns. Selective contribution refers to the fact that some modalities are more important than others for a given task. Diminishing returns refers to redundant modalities which do not improve inferencing.

#### 5.2.2. Strengths

Decision fusion optimizes data processing per modality and has several advantages:

- First, best-fit encodings and latent representations are learned for each modality independently of other modalities. For example, a modality can be modeled using a fine-tuned pretrained LM with a limited number of training epochs (i.e., few shot learning), whereas a different modality can use an ML model over tabular data allowing for a large number of training epochs [100].
- Second, decision fusion is more resilient to incomplete data as the latent representations of the modalities are asynchronous [101].
- Third, modalities are weighed in the decision layer irrespective of the dimension sizes of their latent representations, thereby preventing high dimension modalities from overshadowing low dimension modalities [74].

#### 5.2.3. Limitations

A significant body of evidence is available in the literature to support the superior performance and often better generalizability of decision fusion models or ensemble learners over tabular data [102,103]. However, the following limitations were identified when decision fusion is used with generative learning:

- Decision fusion only allows co-learning in the decision layer after the latent representation of each modality has been established [78].
- Introducing temporal context across the modalities in decision fusion may nullify the resilience of this architecture to irregular and missing data [95,96,104].

### 5.3. Representation Learning Fusion

Representation learning fusion (Figure 1c) is a hybrid of both encoding and decision fusions. It uses encoding fusion on subsets of EHR modalities and later applies decision fusion across the subsets [12,81].

#### 5.3.1. Examples

Representation learning fusion with stacking decisions for EHR data is exemplified in Clinical MAG [68]. The multi-modal adaption gate (MAG) was used to oversee the combination of time-invariant encodings, time-series encodings, and free text. Time series data were encoded using either LSTM, CNN, or a transformer. Clinical notes were encoded with ClinicalBERT [39]. For alignment purpose, one of the modalities is selected as the reference modality. The decision layer is constructed using a softmax or a sigmoid layer over the fused latent representation. The results of this study indicate that uni-modal encodings and their predictive importance are task-dependent.

MedM-PLM is another example of RL fusion. It implements representation learning fusion through cross-attention [77]. Two transformers are used to fuse disease and medications codes with unstructured clinical notes. The first (structured) transformer generates an ontology-enhanced intermediate latent representation of disease and medication codes. The second (unstructured) transformer generates a latent representation of the clinical notes. MedM-PLM uses the same architecture as BERT with an input embedding consisting of a token embedding, a position embedding representing the sequence of the tokens, and an encounter identifier embedding that differentiates between consecutive encounters. The two transformers, structured and unstructured, each develop a CLS embedding which represents the pooling of all hidden layer embeddings for their respective input. During training, these CLS embeddings are swapped between the two transformers to form a joint latent representation of the input. Task specific classification layers are then utilized for fine-tuning and evaluation.

MedM-PLM was shown to outperform baseline models over multiple tasks including medication recommendations, readmission prediction, and ICD coding. For instance, a 5% increase in AUC was reported for readmission prediction over both Med-BERT^1^ [34] and Clinical BERT [39]. Higher performance was also observed compared to a decision fusion over the structured and unstructured latent representations.

#### 5.3.2. Strengths

The main advantage of representation learning fusion is the ability to use co-learning with varying subsets of the EHR modalities. This fusion architecture is also less impacted by irregular and missing data compared to encoding fusion.

#### 5.3.3. Limitations

Engineering the most impact-full fusion path is a major challenge for representation learning fusion. Most fusion paths are handcrafted [68,77]. MUFASA automates fusion path selection using a tournament approach [67], which is computationally intensive.

## 6. Discussion

The reviewed studies demonstrate the potential of multi-modal fusion architectures over EHR data in enhancing clinical decision support (Table 4). Unfortunately, guidelines and methodologies for selecting the adequate encoding and fusion architecture for a given outcome and a clinical setting are unavailable. In fact, even the definition of modality and the classification of fusion architectures are subject to significant variances in the literature.

EHR data encompasses temporal, semantic, and medical concepts. The transformation of these data into modalities should take into consideration the source, encoding, and concept level. Multi-modal architectures should also be organized according to the layer where the fusion occurs, thereby separating learning from fusion constructs.

The present scoping review introduced a revised classification which consists of three fusion architectures: encoding, decision, and representation layer fusions. The main advantages of encoding fusion are co-learning and temporal encoding [20,22,24]. However, this fusion architecture is susceptible to missing data and requires intricate dimension reduction techniques, especially for extended observation periods.

Decision fusion is better adapted for tabular data and ML models [102,103]. It allows each modality to be processed independently making it non-susceptible to missing and irregular data. However, this architecture offers limited co-learning.

The advantages of representation learning fusion are reduced susceptibility to missing data compared to encoding fusion and selective co-learning compared to decision fusion. However, engineering the best fusion path is a difficult problem [67]. Moreover, co-learning techniques that are able to process intermediate temporal, semantic, and concept information are limited.

Future research should consider structured fusion architectures that encourage re-use across multiple clinical applications. These architectures can be constructed from basic building blocks of pretrained encodings and latent representations which can be fine-tuned and assembled to deliver a meta-model for a target outcome. For instance, temporal and semantic encodings can be learned in the encoding layer while co-learning can be introduced in the representation layer (Figure 1). The success of this approach relies on generalizable building blocks for different modalities and different clinical settings.

Pretrained encoding LMs for a few of the EHR modalities such as disease codes (Table 2) and clinical notes (Table 3) currently exist and are being re-used for various clinical applications [13]. For other modalities, such as clinical variables and medications, pretrained LMs are unavailable. Moreover, some of the pretrained LMs consider temporal information (e.g., Med-BERT^1^ [34]), while others do not (e.g., ClinicalBERT [39]). For alignment purposes, encodings must capture temporal information. In addition, a time-aware decay function [95] which can adjust the weights of the encounters according to their relevance to the clinical outcome will also enhance re-use across clinical settings.

Latent representation blocks can be combined to promote co-learning using techniques such as cross-attention [105,106] during fine-tuning. The representation learning layer also represents an ideal opportunity for domain knowledge infusion and modality pruning using techniques such as the ones introduced in [35,80,107,108].

Finally, it is still unclear when DL models with their ability to leverage temporal and semantic information outweigh ML models over tabular data [102,103,109]. Combining these two modeling approaches, potentially in the decision layer, merits further analysis. This may provide a quick pathway for fine-tuning the resulting meta-model to a local patient distribution or a specific target outcome.

## 7. Conclusions

The availability of EHR data opened up the opportunity for the development of ML and DL models which can support diagnosis, prognosis, and general clinical decision support. Several successful models over a single or multiple EHR modalities have been proposed in the literature. Some studies further indicate that fusion models and in particular encoding fusion and representation layer fusion models outperform models trained on a single EHR modality. However, guidelines for how to best encode and combine these modalities while retaining their inherent temporal and semantic information are lacking. Future research should consider techniques that allow meta-models to be constructed from re-usable, pretrained models adapted to different clinical settings in order to reduce the substantial effort needed to hand-craft these models for various clinical outcomes.

## Figures and Tables

**Table 1.** Routine care EHR representative citations and their classification by subject area.

Subject	References
Reviews	[1–7,9–14,18–25]
Taxonomy	[26–31]
Uni-modal Models	[32–49]
Multi-modal Models	[50–83]

**Table 2.** Example uni-modal language models for ICD codes.

Model	Ref.	Year	Training Dataset	Evaluation Task
CONTENT	[32]	2018	CHF	Readmission
TeSAN	[33]	2019	MIMIC-III, CMS	Phenotyping
Med-BERT^1^	[34]	2021	CHF, Truven Health MarketScan	Phenotyping
SG-Co	[35]	2021	KPMAS	Phenotyping, Mortality, Readmission
Medretriever	[36]	2021	Real-world health insurance claim data	Phenotyping
RareBERT	[37]	2021	Symphony Health’s IDV	Phenotyping

**Table 3.** Example of generative encodings for clinical notes.

Model	Ref.	Year	Training Dataset	Evaluation Task
MSATT-KG	[38]	2019	MIMIC-III	ICD coding
Clinical BERT	[39]	2019	MIMIC-III	NER, inferencing
BioBERT	[40]	2020	PubMed	NER, RE, QA
EHR2Vec	[41]	2020	Private EHR	Phenotyping
DCAN	[42]	2020	MIMIC-III	ICD coding
LAAT	[43]	2020	MIMIC-III	ICD coding
HAN	[44]	2020	MIMIC-III	Mortality
Med-BERT^2^	[45]	2021	CMeEE, CMR	NER
MDBERT	[46]	2022	MIMIC-III	ICD coding
GatorTron	[47]	2022	Private EHR, PubMed, Wikipedia	NER, RE, QA, Inferencing
Bioformer	[48]	2023	PubMed	NER, RE, QA, DC
AD-BERT	[49]	2023	Private EHR	Phenotyping

NER: name entity recognition, RE: relation extraction, QA: question answering, DC: Document Classification.

**Table 4.** Representative routine care EHR fusion models.

Model	Ref.	Year	Fusion	Modalities	Dataset	Evaluation Task
Deepr	[50]	2016	Encoding	Structured, Clinical Notes	Private EHR	Mortality, LOS, Phenotyping, Readmission
SAnD	[51]	2018	Encoding	Time Series	MIMIC-III	Mortality, LOS, Phenotyping
Health-atm	[52]	2018	Encoding	Structured	Private EHR, EMRbots	Phenotyping
AXCNN	[53]	2018	RL	Structured, Time Series	Private EHR	Readmission
HA-BiRNN	[54]	2018	RL	Structured, Diagnosis reports	Private EHR	Phenotyping
Patient2vec	[55]	2018	Encoding	Structured	Private EHR	Readmission
MSAM	[56]	2019	RL	Structured	MIMIC-III, Private EHR	Phenotyping
ATTAIN	[57]	2019	Encoding	Structured, Time series	Private EHR	Phenotyping
HCET	[60]	2020	RL	Structured, Clinical Notes	Private EHR	Phenotyping
BEHRT	[61]	2020	Encoding	Structured	CPRD	Phenotyping
HIN	[62]	2020	RL	Structured, Clinical Notes	MIMIC-III	Phenotyping, Symptoms Classification
MM-HCR	[63]	2020	Decision	Clinical Notes, Time Series	MIMIC-III	Mortality
MHM	[64]	2020	RL	Structured, Time Series	MIMIC-III	Phenotyping
BRLTM	[65]	2021	Encoding	Structured, Clinical Notes	Private EHR	Phenotyping
CEHR-BERT	[66]	2021	RL	Structured	CUIMC-NYP	Phenotyping
MUFASA	[67]	2021	Any	Any	MIMIC-III	Phenotyping
Clinical MAG	[68]	2021	RL	Structured, Clinical Notes	MIMIC-III	Phenotyping
EDisease	[69]	2021	RL	Structured, Clinical Notes	Private EHR, NHAMCS	Phenotyping
DeepEMC^2^	[70]	2021	RL	Structured, Clinical Notes	Private EHR	Emergency Risk Classification
TADEL	[71]	2021	Encoding	Structured	MCD	Readmission
LDAM	[72]	2021	RL	Clinical Notes, Time Series	MIMIC-III	Phenotyping
MixEHR-Guided	[73]	2022	RL	Structured, Clinical Notes	MIMIC-III, PopHR	Phenotyping
HAIM	[74]	2022	Decision	Any	MIMIC-III	Mortality, LOS, Phenotyping
Hi-BEHERT	[75]	2022	RL	Structured	CPRD	Phenotyping
MBERT	[76]	2022	RL	Structured, Clinical Notes	MIMIC-III	Mortality
MedM-PLM	[77]	2022	RL	Structured, Clinical Notes	MIMIC-III	Medication Recommender, Readmission, ICD Coding
MCDP	[78]	2022	RL	Structured, Times series	MIMIC-III, MIMIC-IV	Phenotyping, Mortality
DeepBiomarker	[79]	2022	Encoding	Structured	UPMC	Phenotyping
KG-MTT-BERT	[80]	2022	RL	Structured, Unstructured	Private EHR	Phenotyping
ExMed-BERT	[82]	2023	Encoding	Structured	IBM Explorys Therapeutic dataset	Phenotyping
TransformerEHR	[83]	2023	Encoding	Structured	Private EHR	Phenotyping

## Abbreviations

ATC: Anatomical Therapeutic Chemical
BERT: Bidirectional Encoder Representation from Transformers
DC: Document Classification
GPI: Generic Product Identifier
ICD: International Classification of Diseases
LM: Language Model
LOS: Length of Stay
NER: Named Entity Recognition
QA: Question Answering
RE: Relation Extraction
RL: Representation Learning
